# Transfer Learning-Based Model for Diabetic Retinopathy Diagnosis Using Retinal Images

**DOI:** 10.3390/brainsci12050535

**Published:** 2022-04-22

**Authors:** Muhammad Kashif Jabbar, Jianzhuo Yan, Hongxia Xu, Zaka Ur Rehman, Ayesha Jabbar

**Affiliations:** 1 Faculty of Information Technology, Beijing University of Technology, Beijing 100124, China; kashif.superior@gmail.com (M.K.J.); yanjianzhuo@bjut.edu.cn (J.Y.); 2 Department of Computer Science and IT, Gujrat Campus, The University of Lahore, Gujrat 50700, Pakistan; zaka.rehman.uol@gmail.com; 3 Department of Science & Technology, University of Education, Lahore 54770, Pakistan; ayeshajabbar009@gmail.com

**Keywords:** diabetic retinopathy, annotated data insufficiency, transfer learning, fundus images, computer-aided diagnosis, convolutional neural network

## Abstract

Diabetic retinopathy (DR) is a visual obstacle caused by diabetic disease, which forms because of long-standing diabetes mellitus, which damages the retinal blood vessels. This disease is considered one of the principal causes of sightlessness and accounts for more than 158 million cases all over the world. Since early detection and classification could diminish the visual impairment, it is significant to develop an automated DR diagnosis method. Although deep learning models provide automatic feature extraction and classification, training such models from scratch requires a larger annotated dataset. The availability of annotated training datasets is considered a core issue for implementing deep learning in the classification of medical images. The models based on transfer learning are widely adopted by the researchers to overcome annotated data insufficiency problems and computational overhead. In the proposed study, features are extracted from fundus images using the pre-trained network VGGNet and combined with the concept of transfer learning to improve classification performance. To deal with data insufficiency and unbalancing problems, we employed various data augmentation operations differently on each grade of DR. The results of the experiment indicate that the proposed framework (which is evaluated on the benchmark dataset) outperformed advanced methods in terms of accurateness. Our technique, in combination with handcrafted features, could be used to improve classification accuracy.

## 1. Introduction

Diabetic retinopathy is directly associated with the prevalence of diabetes, which is now at epidemic proportions worldwide [1]. Currently, about 463 million diabetes patients are present, and approximately one-third of them have some form of diabetic retinopathy [2]. The international diabetes federation (IDF) reported that diabetes patients are expected to reach about 552 million by 2035 and 642 million by 2040 [3,4]. There are more than 158.2 million people currently suffering from DR, and this number is estimated to increase to about 191 million by 2030 [5].

**Figure 1:**
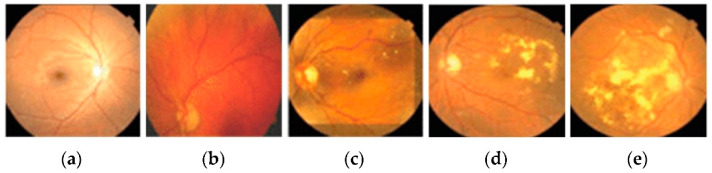
These images show the different types of retinopathies in the fundus images. (**a**) Normal, (**b**) mild, (**c**) moderate, (**d**) severe, and (**e**) proliferative.

DR is an ocular impediment caused by diabetic disease; it has held its position as one of the main factors behind the occurrence of blindness globally [6,7,8]. DR develops because of the long-standing occurrence of diabetes mellitus. The risks of disease are more common in patients with uncontrolled blood sugar. Generally, DR develops gradually and may not cause any symptoms or only mild vision loss in the primary stages. Eventually, if treatment and diagnosis are not performed in a timely manner, then it tends to cause blindness [9]. DR is generally categorized into three categories, normal, non-proliferative DR (NPDR), and proliferative DR (PDR), based on the progression of the diabetic retinopathy [10]. NPDR develops when new retinal blood vessels do not grow, and the blood vessel walls become faded. NPDR is divided further into mild, moderate, or severe stages of disease. In PDR, the retinal area is occupied by new blood vessels and obstructs the blood supply to the retina. Figure 1 shows samples of NPDR, PDR, and its subdivided classes of retinopathy discussed above [11].

DR detection and grading at initial periods is laborious, time-intensive, and requires domain experts [12]. Moreover, the manual screening of DR patients indorses ex- tensive inconsistency among various clinicians. Approximately 79% of DR patients belong to underdeveloped or developing nations, which are deficient in ophthalmologists and a basic setup for DR detection [13]. With the rapid prevalence of DR worldwide, manual screening techniques are unable to keep pace with the demand for diagnosis methods [14].

Due to the developments in computer vision techniques, numerous automatic techniques have been projected by researchers for the diagnosis of DR. There are various challenges associated with enhancements in computer-aided diagnosis (CAD) systems, such as identification of lesions from a retinal image, subdivision of optic disc, segmentation of blood vessel, etc. [15,16]. Although machine learning-based systems have shown resilient performance in DR detection, their efficacy is highly dependent on handcrafted features, which are very difficult to generalize [17]. To overcome such limitations, deep learning (DL) methods provide automatic feature extraction and classification from fundus images. The major determination of this study is to explain an efficient method for categorizing early-stage DR to assist ophthalmologists. The key contribution of this study is highlighted in the following points:

1. A VGGNet model based on transfer learning is proposed for detecting and classifying diabetic retinopathy.
2. Implementation of various preprocessing techniques such as interpolation image resizing, weighted Gaussian blur, and CLAHE for improving the value and visibility of retinal images.
3. Performed data augmentation operations on each grade of DR individually to overcome annotated data insufficiency and to make a balanced dataset.
4. A comprehensive DR classification system is developed with accuracy and robustness.
5. Evaluation of the proposed model is performed on a large dataset, EyePACS, with 35,126 retinal fundus images.
6. Various performance measures are implemented, such as accuracy, sensitivity, specificity, and the AUC to verify the analytical skill.

The paper is compiled in the following manner: The dataset details and the proposed framework is elaborated in Section 2. Results and Discussions are described in Section 3 and the Conclusions in Section 4.

## 2. Methodology

**Figure 2:**
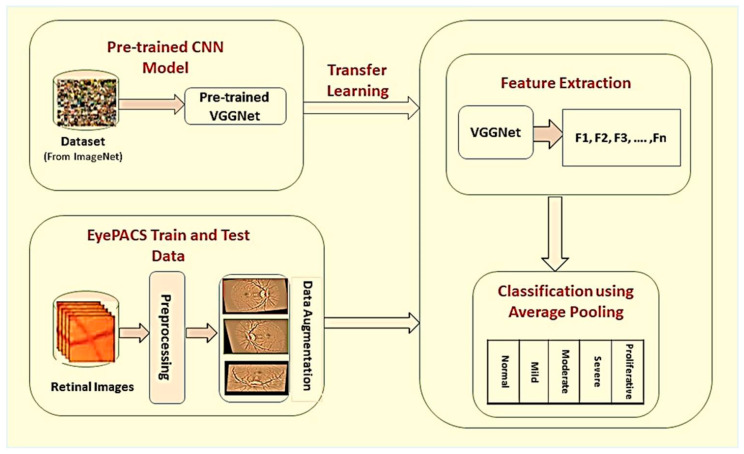
Proposed framework for detection and classification of diabetic retinopathy. In the first phase, retinal images are preprocessed, and data augmentation operations are performed individually on each grade of DR to improve classification accuracy. In the last, model-based on transfer learning is used for automatic features extraction and classification of DR into different stages.

The proposed framework of DR classification was mainly categorized into the following steps: image preprocessing, data augmentation, feature extraction, and classification. The graphical illustration of the proposed work is presented in Figure 2.

### 2.1. The Kaggle EyePACS Dataset

The public dataset EyePACS was used for acquiring fundus images of the retina via Kaggle.com (accessed on 24 March 2021). These images were labeled by the ophthalmologists and subdivided into five categories: normal, mild, moderate, severe, and proliferative DR, as shown in Figure 1. The details related to this dataset are stated in Table 1.

### 2.2. Image Preprocessing

The EyePACS dataset is a heterogeneous dataset containing images from various smaller datasets captured with different cameras, under different adjustments, of different sizes, and with a lot of brightness and illumination differences; therefore, we adopted various preprocessing steps to standardize these images. First, we resized the fundus images to a uniform size by using bicubic interpolation over a 4 × 4-pixel neighborhood. We preferred this interpolation over simple resizing because it resizes the image by sustaining quality and locking the aspect ratio.

Generally, the retinal images were yellowish with a dark background. The fundus details did not overlap with the background and thus can be abolished to reduce noise. Equalizing the black background of the fundus images resulted in darkness being expanded into the image’s details [18]. Concerning this matter, we agreed with preprocessing to delete the black background by setting the pixel value to zero and non-zero for all bright regions. After thresholding was performed, the extraction of the green channel was performed. This green channel conserves more retinal data in comparison to the red or blue channels. The implementation of CLAHE, which is the contrast limited adaptive histogram equalization, took place for improving the quality of the retinal image as well as for enhancing the small areas. 

Then, the weighted Gaussian blur was applied to images to reduce noise and increase image structure [19]. The mathematical expression to calculate the Gaussian function in two dimensions (*x*, *y*) along with *σ* standard deviation is given in Equation (1).

(1) $$G(x,y)=\frac{1}{2\pi \sigma^{2}}\epsilon^{\frac{x^{2}+y^{2}}{2\sigma^{2}}}$$

### 2.3. Data Augmentation

**Figure 3:**
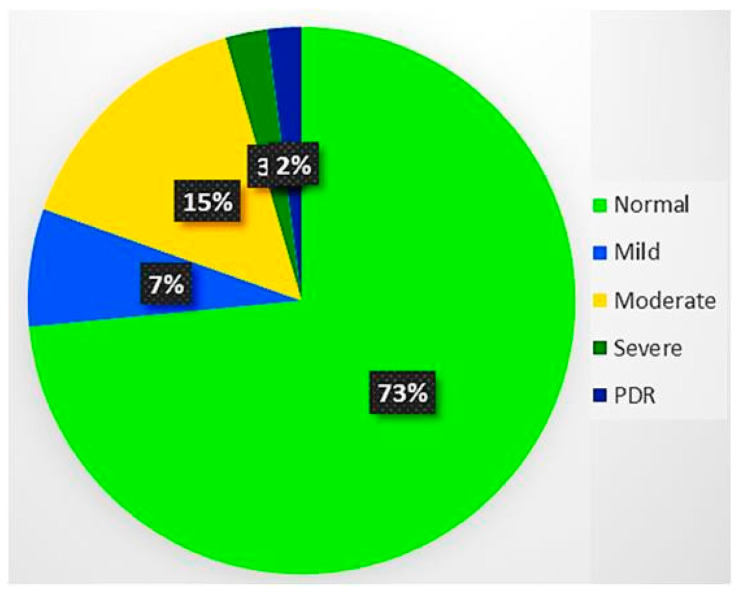
Dataset distribution over DR severity. There were about 73% of images in the normal category, while only 2% of them from the proliferative DR category. Thus, it was an imbalanced dataset with 36:1 for normal and proliferative DR.

The training dataset size is one of the key features for the efficient performance of the DL models. Therefore, it is mandatory to have a larger dataset for the training of deep learning architecture to prevent generalization and overfitting issues. Although the Kaggle EyePACS dataset size is sufficient, it is considered very small as compared to the ImageNet [20] dataset. The dataset distribution over the classes was highly imbalanced, as shown in Figure 3, where the maximum images were from grade 0. This highly imbalanced dataset was in the ratio of (36:3:7:1:1), which may cause incorrect classification. We performed data augmentation operations to amplify the retinal dataset at various scales and to eliminate the noise in fundus images.

**Figure 4:**
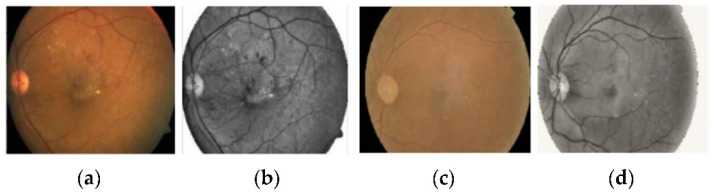
The preprocessed retinal images after applying contrast limited adaptive histogram equalization to adjust contrast in images. (**a**,**c**) Original retinal fundus images, (**b**,**d**) preprocessed images.

We employed various data augmentation operations on each grade differently because of the highly imbalanced nature of the dataset. The visual exemplification of some augmentation techniques performed on preprocessed images is given. This augmentation operation includes cropping, flipping, translating, shearing, rotating, zooming, Gaussian scale-space theory (GST) augmentation [21], and Krizhevsky augmentation [22]. To produce visually appealing images, the clipping limit of CLAHE was set to 2 along the tile grid and 8, as shown in Figure 4.

**Figure 5:**
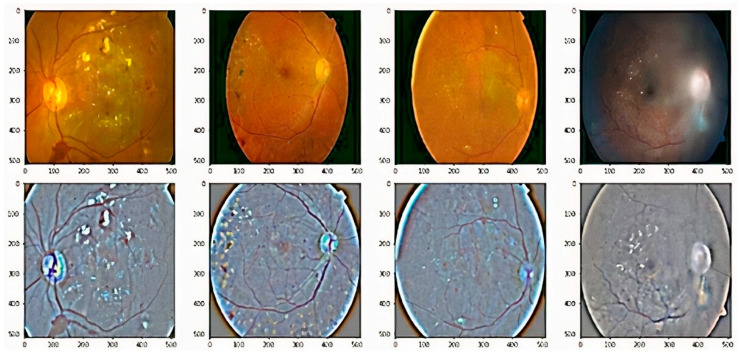
Some examples of adding weighted Gaussian blur to the retinal images, which is employed to reduce noise and increase image structure. First row are original fundus images, and second images are output of the preprocessed images.

For improvement of image quality and enhancement of image structure, we imployed weighted gaussian blur to the retinal fundus images. Figure 5 shows the effects of gaussian blur on the retinal fundus images. The representation of images before and after applying this method of image processing is shown in the Figure 5.

Cropping: Images were randomly cropped from the corner and center to 60–75% of the original image.

Flipping: Images were flipped on both the *X* and *Y* axes.

Translating: Images were shifted between 0 and 30 pixels.

Shearing: Images were sheared between 0 and 180 degrees randomly. 

Rotating: Images were rotated randomly in the range of 0 to 360 degrees. 

Zooming: Images were zoomed in the range of (0.7, 1.3).

GST Augmentation: The GST-based augmentation was performed for a two-dimensional image.

Krizhevsky Augmentation: Krizhevsky color augmentation technique was used for dataset augmentation.

**Figure 6:**
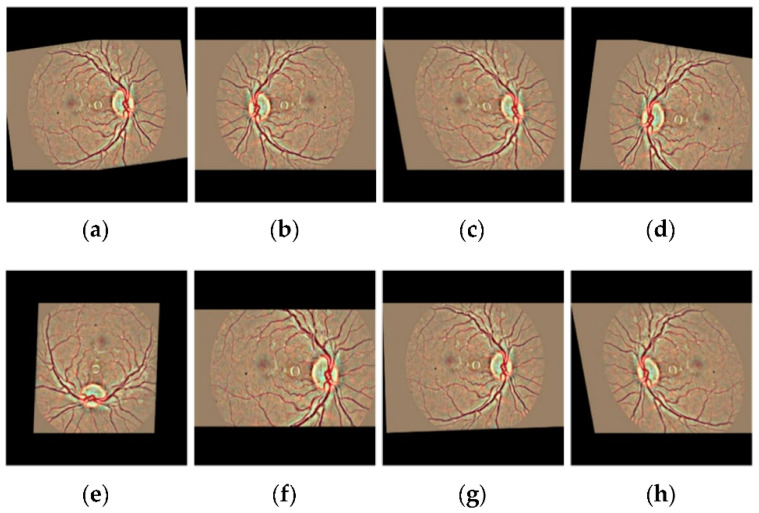
The visual exemplification of some augmentation operations performed on preprocessed images to augment the retinal dataset (**a**) Original image (**b**) Cropping (**c**) Shearing (**d**) Flipping (**e**) Rotating (**f**) Zooming (**g**) Translating (**h**) All augmentation.

The representation of images after applying various data augemntation operation is displayed in the Figure 6.

The details of augmentation operations after applying them to the training dataset are given in Table 2. Different augmentation operations were performed on each grade of fundus images. The augmented dataset was 3.6 times greater than the original dataset and was highly balanced with 1:1 for all grades of DR.

### 2.4. Proposed Architecture

Although deep learning algorithms are good enough to solve various classification problems, the main problem with the classification of medical images is the unavailability of labeled data. Transfer learning is widely adopted to overcome annotated data insufficiency by reusing already trained deep convolutional neural networks for another identical task. Thus, it may be employed for reducing the training overhead as well as for training with a smaller dataset. There arise questions about the implementation of transfer learning to overwhelm annotated data availability in medical image classification based on deep learning. In this study, we opted for pre-trained VGG 16 to take advantage of fixed size 3 × 3 kernel filters for detection.

The architecture of the proposed model is shown in Table 3. This architecture consists of 16 layers with an input layer is 512 × 512. A 3 × 3 kernel size filter, united bias, and stride of 1 were utilized for all convolutional layers other than the first layer with a stride of 2. A 2 × 2 kernel size filter along with a stride of 2 was selected for all the max-pooling layers; extracted features were flattened before forwarding to the connected layers. The usage of the activation function rectified linear unit (ReLU) was made in all layers, and a drop of 0.5 was applied before the first two fully connected layers to evade overfitting. After convolution layers, two fully connected layers were added, with each having 1024 neurons. Finally, a softmax function was used as a single neuron output layer for the early-stage detection and classifying the diabetic retinopathy.

### 2.5. Training

Training of transfer learning-based VGGNet was performed through the process of fine-tuning the hyperparameters. The model was trained through Adam’s optimization function, and the learning rate was set to 0.0001. The weights of the network were initialized randomly through 32 batch-sizes and then trained on a network for 300 epochs. The momentum size was set at 0.9, and categorical cross-entropy was considered as an objective function. Data augmentation operations were performed separately on each grade to overcome data misbalancing issues. The details of these hyperparameters are given in Table 4.

## 3. Results and Discussion

By utilizing a transfer learning technique, a detailed analysis of the working of the proposed scheme for diabetic retinopathy was evaluated. The Kaggle EyePACS dataset after data augmentation operations were segmented into 80%, 20% for the training and testing datasets. 

### 3.1. The System Configurations

A system with the following specifications was used for the methodology.

OS: Linux Mint 18.1 operating system.

CPU: Intel(R) 3rd Generation Core (TM) i5-3470 CPU @ 3.20 GHz @ 3.20 GHz;

CPU Ram: 16 GB;

GPU: Quadro K620;

Graphics card Ram size: 12 GB.

### 3.2. The Performance Metrics

For evaluation of the overall working of the presented scheme, we analyzed the performance of abnormal human activity recognition from a confusion matrix [23] and then computed the following performance metrics.

Precision: Expressed as the ratio of a total number of *TP* with respect to the total number of component tags as appropriate to the positive class (i.e., the sum of *TP* and *FP*). Positive Predictive Value (PPV) is used to denote the precision. Precision can be measured as follows:

(2) $$\mathrm{Precision}=\frac{TP}{TP+FP}$$

F1 Score: The harmonic mean of recall and precision is used to measure it as in Equation (3):

(3) $$F1\;\mathrm{Score}=\frac{2\times \mathrm{Precision}\times \mathrm{Recall}}{\mathrm{Precision}+\mathrm{Recall}}$$

Accuracy: The ratio of a number of components *TP* and *TN* to the total number of components *TP*, *TN*, *FP*, and *FN* is used to measure the accuracy. The accuracy can be calculated using the following Equation (4).

(4) $$\mathrm{Accuracy}=\frac{TP+FN}{TP+FN+TN+FP}$$

Specificity: It can be expressed in the ratio of the number of *TN* with respect to the total number of components that are appropriate to the negative class (i.e., the sum of *TN* and *FP*). The mathematical expression is shown in Equation (5).

(5) $$\mathrm{Recall}=\frac{TN}{TN+FP}$$

The Area Under the Curve: It can be obtained by performing a definite integral between the two points. This can be measured by using the following mathematical Equation (6).

(6) $$\mathrm{Area}\;\mathrm{Under}\;\mathrm{the}\;\mathrm{Curve}=\frac{1}{2}\left(\frac{TP}{TP+FN}+\frac{TN}{TN+FP}\right)$$

### 3.3. Result Analysis

After the training of the proposed model, the learning data was passed to the combined extracted features by using transfer learning. Besides the proposed VGGNet approach, different transfer learning models were also implemented, as shown in Table 5.

From Table 5, we can make observations that the ResNet, GoogLeNet, and AlexNet gave average accuracies of 92.40%, 93.75%, and 94.62%, respectively. Meanwhile, the proposed model stated an accuracy level of about 96.61%. Thus, it is an efficient method for detecting and classifying diabetic retinopathy. Furthermore, the data is split into the training and testing data. For training purposes, 80% of the data were used; for testing the proposed architecture, the rest of the 20% of unseen data were used. The proposed model was assessed through both the original and augmented dataset, as presented in Table 6.

**Figure 7:**
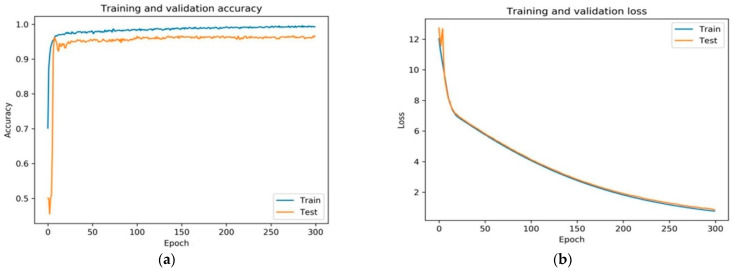
(**a**) Shows the training and validation accuracy of the proposed framework and (**b**) shows the training and validation loss of the proposed fine-tuned VGGNet framework.

The proposed model showed higher accuracy for detecting and classifying diabetic retinopathy on a balanced augmented dataset. In Figure 7a,b, it can be observed that the training plots and validation accuracy improved constantly.

The fine-tuned version of VGGNet displayed exceedingly acceptable performance. The rate of training and validation accuracy continuously improved with an increase in each epoch. The loss curve indicates that the loss of training and validation decreased with each epoch.

The implemetation of various data augmentation techniques, the augmented data was obtained. The acquired augmented data was distributed into training and testing data as shown in the Table 7.

In addition to average results, the best performance results were also important and retained in this paper because the validation accuracy can be altered with each epoch during the CNN training. Table 8 shows the prevalence of the disease on the basis of the diagnosis test.

The comparative analysis of the proposed architecture was carried out with five up-to-date methods to relate its strength, as shown in Table 9.

**Figure 8:**
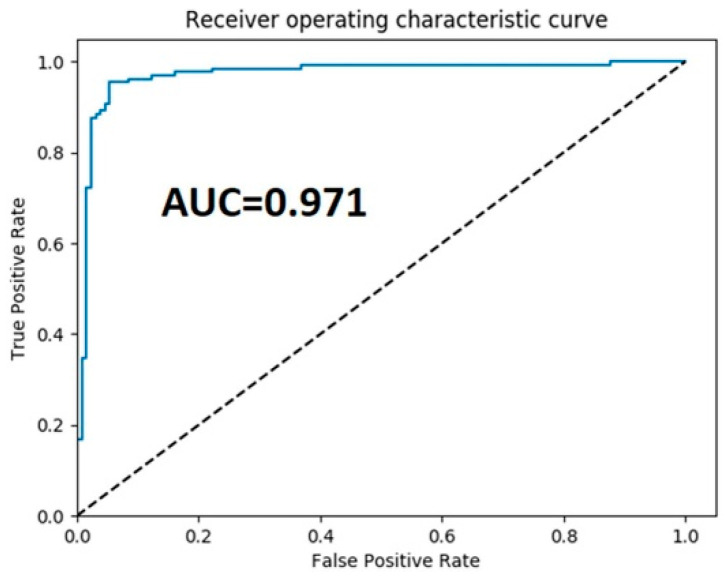
The receiver operator characteristics curve of the proposed framework.

Observations made by the methods in [24,25,26,27,28,29,30,31,32,33,34,35] gave average accuracies of 82.3, 85.3, 90.4, 78.7, 95.68, 95.03%, and so on, as shown in the figure. This method yielded an accuracy of 96.6%, which is much higher than baseline methods. The experimental outputs indicate the strength of the proposed architecture with respect to the accuracy as compared to existing methods. Figure 8 shows the receiver the operating characteristic (ROC) and AUC curve of the proposed model.

## 4. Conclusions

We proposed a framework for the automatic detection and classification of diabetic retinopathy by using the transfer learning concept in this paper. For the preprocessing, we employed effective preprocessing techniques such as interpolation image resizing, weighted Gaussian blur, and non-local mean denoising (NLMD) for improving the visibility of an image. After preprocessing, transfer learning-based VGGNet architecture was used for classifying DR into the normal, mild, moderate, severe, and proliferative classes. The experimental studies were conducted on the Kaggle EyePACS public dataset. The effectiveness of the method was evaluated using numerical procedures such as the sensitivity, specificity, accuracy, and area under the curve.

We explored the extraction of features in fundus images using VGGNet architecture and united using the concept of transfer learning to improve classification performance. Moreover, we also employed various data augmentation operations on each grade of DR differently to make a balanced dataset and improve the efficiency of architecture. Finally, the results of the proposed study were compared to many deep learning architectures, and a comparison was made with the baseline methods. It is concluded that this proposed method displays exceptional presentation regarding various statistical measures. In the future, we intend to use hand-engineered features along with CNN to further improve classification accuracy.

## Figures and Tables

**Table 1:** Kaggle EyePACS dataset details.

Grade	Severity	No. of Images	% of Total Images
0	Normal	25,810	73.15
1	Mild	2443	6.96
2	Moderate	5292	15.07
3	Severe	873	2.81
4	PDR	708	2.01
Total		35,126	100%

**Table 2:** The dataset statistics by using data augmentation operations.

Grade	Severity	No. of Images	Operations	Augmented Images
0	Normal	25,810	0	25,810
1	Mild	2443	11	24,430
2	Moderate	5292	5	26,460
3	Severe	873	29	25,317
4	PDR	708	36	25,488
Total		35,126		127,505

**Table 3:** The proposed architecture for early-stage detection and classification of diabetic retinopathy comprising of 16 layers.

Sr. No.	Layer Type	Kernel Size and Number	Stride	Output
1	input	—	—	(512,512,3)
2	conv 1_1	3 × 3 × 32	2	(256,256,32)
3	conv 1_2	3 × 3 × 32	1	(255,255,32)
4	max-pooling	2 × 2	2	(127,127,32)
5	conv 2_1	3 × 3 × 64	1	(63,63,64)
6	conv 2_2	3 × 3 × 64	1	(64,64,64)
7	max-pooling	2 × 2	2	(32,32,64)
8	conv 3_1	3 × 3 × 128	1	(32,32,128)
9	conv 3_2	3 × 3 × 128	1	(33,33,128)
10	conv 3_3	3 × 3 × 128	1	(34,34,128)
11	max-pooling	2 × 2	2	(17,17,128)
12	conv 4_1	3 × 3 × 256	1	(9,9,256)
13	conv 4_2	3 × 3 × 256	1	(10,10,256)
14	conv 4_3	3 × 3 × 256	1	(11,11,256)
15	max-pooling	2 × 2	2	(6,6,256)
16	conv 5_1	3 × 3 × 512	1	(6,6,512)
17	conv 5_2	3 × 3 × 512	1	(5,5,512)
18	conv 5_3	3 × 3 × 512	1	(4,4,512)
19	max-pooling	2 × 2	2	(2,2,512)
20	fully connected	1024	—	−1024
21	fully connected	1024	—	−1024
22	fully connected	1	—	−1

**Table 4:** Diagnostic test for the prevalence of the disease.

Sr. No.	Hyperparameters	Value
1	Learning Rate	0.0001
2	Batch Size	32
3	Activation Function	ReLU
4	Epochs	300
5	Optimizer	Adam
6	Momentum	0.9
7	Loss Function	Categorical Cross-Entropy

**Table 5:** The performance comparison of the proposed model on an original and augmented dataset.

Sr. No	Architecture	Sensitivity	Specificity	Accuracy	Precision	F1-Score	AUC
1	ResNet	0.854	0.943	0.924	0.977	0.918	0.924
2	GoogLeNet	0.895	0.989	0.937	0.99	0.939	0.935
3	AlexNet	0.953	0.938	0.946	0.937	0.945	0.949
4	VGGNet	0.949	0.984	0.966	0.985	0.967	0.971

**Table 6:** The performance comparison of the proposed model and different existing transfer learning models on an original and augmented dataset.

Sr. No.	Dataset	Accuracy	AUC
1	Original	0.936	0.954
2	Augmented	0.966	0.971

**Table 7:** The distribution of augmented data into training and testing data.

Sr. No.	Grade	Severity	Original Images	Augmented Images	Training	Validation
1	0	Normal	25,810	25,810	20,648	5162
2	1	Mild	2443	24,430	19,544	4886
3	2	Moderate	5292	26,460	21,168	5292
4	3	Severe	873	25,317	20,254	5063
5	4	PDR	708	25,488	20,390	5098
Total			35,126	127,505	102,004	25,501

**Table 8:** Diagnostic test for the prevalence of the disease.

	DR	Normal
Identified as having DR	*TP*	*FP*
Identified as having no DR	*FN*	*TN*

**Table 9:** Performance comparison of the proposed framework with baseline methods.

Reference #	Author	Year	Model	Target	Accuracy
[24]	Rakhlin	2018	CNN	DR	85.3
[25]	Sengupta et al.	2019	CNN	DR	90.4
[26]	Gulshan et al.	2016	CNN	DR	91.7
[27]	Chang	2018	CNN	DR	78.7
[28]	Wan	2018	CNN	rDR	95.68
[29]	Gargeya	2017	CNN	DR	95.03
[30]	Zeng	2019	CNN	rDR	82.2
[31]	Zhang	2019	CNN	rDR	87.06
[32]	Lin	2018	CNN	DR	86.10
[33]	Li	2018	DCNN	vtDR	86.04
[34]	Seth	2018	CNN	rDR	84.36
[35]	Keel	2018	Third-party DL algorithm	rDR	89.08
	Proposed Method		CNN	DR	96.6

## Data Availability

The data presented in this study are available on request to the corresponding author.
