# Correction: Jabbar et al. Transfer Learning-Based Model for Diabetic Retinopathy Diagnosis Using Retinal Images. *Brain Sci.* 2022, *12*, 535

**DOI:** 10.3390/brainsci14080777

**Published:** 2024-07-31

**Authors:** Muhammad Kashif Jabbar, Jianzhuo Yan, Hongxia Xu, Zaka Ur Rehman, Ayesha Jabbar

**Affiliations:** 1 Faculty of Information Technology, Beijing University of Technology, Beijing 100124, China; kashif.superior@gmail.com (M.K.J.); yanjianzhuo@bjut.edu.cn (J.Y.); 2 Department of Computer Science and IT, Gujrat Campus, The University of Lahore, Gujrat 50700, Pakistan; zaka.rehman.uol@gmail.com; 3 Department of Science & Technology, University of Education, Lahore 54770, Pakistan; ayeshajabbar009@gmail.com

In the original publication [1], newly added ref. [11] was not cited. The citation has now been inserted in the Introduction, Paragraph 2, and should read:

DR is an ocular impediment caused by diabetic disease; it has held its position as one of the main factors behind the occurrence of blindness globally [6–8]. DR develops because of the long-standing occurrence of diabetes mellitus. The risks of disease are more common in patients with uncontrolled blood sugar. Generally, DR develops gradually and may not cause any symptoms or only mild vision loss in the primary stages. Eventually, if treatment and diagnosis are not performed in a timely manner, then it tends to cause blindness [9]. DR is generally categorized into three categories, normal, non-proliferative DR (NPDR), and proliferative DR (PDR), based on the progression of the diabetic retinopathy [10]. NPDR develops when new retinal blood vessels do not grow, and the blood vessel walls become faded. NPDR is divided further into mild, moderate, or severe stages of disease. In PDR, the retinal area is occupied by new blood vessels and obstructs the blood supply to the retina. Figure 1 shows samples of NPDR, PDR, and its subdivided classes of retinopathy discussed above [11].

The newly added reference [11] appears below, the subsequent reference numbers are also incremented by one:

11. El-Bab, M.F.; Zaki, N.S.; AL-Sisi, A.; Akhtar, M. Retinopathy and risk factors in diabetic patients from Al-Madinah Al-Munawarah in the Kingdom of Saudi Arabia. *Clin. Ophthalmol.* **2012**, *6*, 269–276. https://doi.org/10.2147/OPTH.S27363.

With this correction, the order of some references has been adjusted accordingly. We hereby state that the scientific conclusions are unaffected. This correction was approved by the Academic Editor. The original publication has also been updated.
